# Relationship between *SLC6A2* gene polymorphisms and brain volume in Han Chinese adults who lost their sole child

**DOI:** 10.1186/s12888-023-05467-4

**Published:** 2024-01-02

**Authors:** Zhuoman Xia, Zhihong Cao, Wesley Surento, Li Zhang, Lianli Qiu, Qiang Xu, Longjiang Zhang, Lingjiang Li, Yang Cao, Yifeng Luo, Guangming Lu, Rongfeng Qi

**Affiliations:** 1 Department of Medical Imaging, Jinling Hospital, Affiliated Hospital of Medical School, Nanjing University, Nanjing, Jiangsu 210002 China; 2 Department of Radiology, the Affiliated Yixing Hospital of Jiangsu University, 75 Tongzhenguan Road, Wuxi, Wuxi 214200 China; 3 Imaging Genetics Center, Mark and Mary Stevens Neuroimaging and Informatics Institute, University of Southern California, Marina del Rey, Los Angeles, CA 90292 USA; 4 Mental Health Institute, the Second Xiangya Hospital, Key Laboratory of Psychiatry and Mental Health of Hunan Province, National Technology Institute of Psychiatry, Central South University, Changsha, Hunan 410011 China; 5 College of Arts & Science, Vanderbilt University, Nashville, TN 37235 USA

**Keywords:** Post-traumatic stress disorder, Voxel-based morphometry, *SLC6A2*

## Abstract

**Background:**

Norepinephrine transporter (NET) is encoded by the *SLC6A2* gene and is a potential target for studying the pathogenesis of PTSD. To the best of our knowledge, no prior investigations have examined *SLC6A2* polymorphism-related neuroimaging abnormalities in PTSD patients.

**Methods:**

In 218 Han Chinese adults who had lost their sole child, we investigated the association between the T-182 C *SLC6A2* genotype and gray matter volume (GMV). Participants included 57 PTSD sufferers and 161 non-PTSD sufferers, and each group was further separated into three subgroups based on each participant’s *SLC6A2* genotype (TT, CT, and CC). All participants received magnetic resonance imaging (MRI) and clinical evaluation. To assess the effects of PTSD diagnosis, genotype, and genotype × diagnosis interaction on GMV, 2 × 3 full factorial designs were used. Pearson’s correlations were used to examine the association between GMV and CAPS, HAMD, and HAMA.

**Results:**

The *SLC6A2* genotype showed significant main effects on GMV of the left superior parietal gyrus (SPG) and the bilateral middle cingulate gyrus (MCG). Additionally, impacts of the *SLC6A2* genotype-diagnosis interaction were discovered in the left superior frontal gyrus (SFG). The CAPS, HAMA, and HAMD scores, as well as the genotype main effect and diagnostic *SLC6A2* interaction, did not significantly correlate with each other.

**Conclusion:**

These findings indicate a modulatory effect that the *SLC6A2* polymorphism exerts on the SPG and MCG, irrespective of PTSD diagnosis. We found evidence to suggest that the *SLC6A2* genotype-diagnosis interaction on SFG may potentially contribute to PTSD pathogenesis in adults who lost their sole child.

**Supplementary Information:**

The online version contains supplementary material available at 10.1186/s12888-023-05467-4.

## Introduction

As of January 1, 2016 the “One-Child Policy”, which has been implemented in China for 36 years was abolished, and a “Two-Child Policy” was fully implemented nationwide [1]. Although the “One-Child Policy” successfully provided solutions for its intended population issues, its related problems are also gradually becoming apparent. Among them, losing one’s sole child has become a major public health concern, and there is also increasing scholarly attention towards studying families that have lost the sole child [2–4]. Children are of great value to their parents, providing them with a sense of empowerment and self-worth. For a parent, experiencing the death of a child, especially a sole child, is a traumatic life event that carries long-term ramifications for their physical and mental health. Parents who underwent such loss are called *Shidu* parents; their experience may potentially cause great physical and psychological impairment, and even give rise to the development of post-traumatic stress disorder (PTSD) [5].

PTSD is a heterogeneous mental disorder that occurs and persists after experiencing serious threatening and catastrophic events. It is known to cause adverse psychological effects and serious damage to social function, greatly impacting the life of the patient. Research has shown that about 70% of people will experience traumatic events in their life, with 10% ~ 20% of them eventually developing PTSD as a result of environmental and individual factors [6]. It should be noted that losing one’s sole child does not necessarily cause one to develop PTSD. [7]. Mechanisms that contribute to PTSD development have not been adequately studied and understood. A joint genomics study of tens of thousands of PTSD patients posits that PTSD development is associated with hereditary factors [8]. Candidate genes related to the dopaminergic system, pituitary adrenal axis, hypothalamic serotonergic system, neuroinflammation and other neurotransmitter systems have been studied in PTSD and found to be directly related to its pathogenesis [9].

The norepinephrine (NE) system has wide projections from the locus coeruleus to the cerebral cortex, and is involved in the regulation of various brain functions and behaviors such as arousal, memory acquisition, attention, vigilance, and response to stress [10]. NE is synthesized by the amino acid tyrosine through a sequential reactions catalyzed by tyrosine hydroxylase, DOPA decarboxylase and dopamine β hydroxylase (DβH). NE molecules released from the anterior terminal of synapses bind to different subtypes of adrenergic receptors, triggering a variety of physiological and pharmacological reactions [11]. Extracellular NE can be degraded by enzymes such as monoamine oxidases (MAO) or catechol-O-methyltransferases (COMT), or brought back to the anterior terminal of synapses by norepinephrine transporters (NET) [12]. The NET regulates synaptic norepinephrine signals in the brain and the autonomic sympathetic nervous system, maintaining intracellular norepinephrine reserves [13, 14]. In addition to PTSD, many other health conditions such as hypertension, obesity, anorexia nervosa, ADHD and depression, are related to NET dysfunction [15]. Encoded by the *SLC6A2* gene, the NET is a presynaptic Na^+^/CL^−^ dependent transporter that is distributed in the locus coeruleus, frontal cortex, amygdala, thalamus, hippocampus and cerebellar cortex [16]. The *SLC6A2* gene, solute carrier family 6 member 2, which is found on chromosome 16q12.2, is the most studied gene involving the noradrenergic system [17]. Previous studies have shown an independent association between T-182 C polymorphisms in the *SLC6A2* 5’ flanking promoter region (rs2242446) and PTSD anxiety arousal symptoms [18]. Variability in brain morphology of patients with Genetics and epigenetic factors may play an important role in regulating brain development and neurodegeneration in PTSD. Some studies have demonstrated a link between brain volume and genetic polymorphisms in PTSD, for example, the brain-derived neurotrophic factor (*BDNF*) Val66met polymorphism may increase susceptibility to PTSD and anxiety disorders via an interaction with reduced ventromedial prefrontal cortex and insular cortex volume [19], the FK506-binding protein 5 (*FKBP5*) rs1360780 is associated with smaller gray matter volumes in the dorsal anterior cingulate cortex [20], and the catechol-O-methyltransferase (*COMT*) polymorphism moderates the association between PTSD and hippocampal volume [21]. It is, however, unknown whether the *SLC6A2* polymorphisms contribute to neuroimaging changes in PTSD patients according to previous studies. In this context, we aim to investigate whether there is a certain connection between the *SLC6A2* polymorphisms and objective imaging indicators in Chinese *Shidu* population. We hypothesized that, the *SLC6A2* polymorphisms moderated the association between PTSD diagnosis and GMV.

## Materials and methods

### Participants

Participants in this study were recruited through advertisement from a PTSD survey of Han Chinese parents in Jiangsu Province, China, who had lost their sole child, from September 2016 to March 2017. The reasons of losing only child include traffic accident, accidental explosion, suicide, cancer, sudden death and so on. An ethics committee at the Medical Research Ethics Committee of Jiangsu University approved this study. In total, 237 Han adults who had lost their sole child took part in the study. Informed consent was obtained from all participants prior to participating in the study. With prior major traumatic exposures as an exclusion criterion, the participants were successfully interviewed and screened by the clinician-administered PTSD scale (CAPS). CAPS is a structured interview that uses standardized questions to diagnose and assess the severity of PTSD. It contains 30 items in total, including seventeen core symptoms and eight related symptoms, with three subscales of repeated experience, avoidance and increased alertness. The higher the score of each symptom, the greater the severity of the symptom. While if the total score is less than nineteen, it means asymptomatic. To confirm the diagnoses of PTSD and other potential psychiatric comorbidities, the Chinese version of the structured clinical interview for DSM-IV58(SCID) [22] —revised by Prof. Lipeng Fei from the Beijing Hui Long Guan Hospital—was used to screen all 237 adults. Among these participants, there were 57 trauma-exposed adults diagnosed with PTSD (19 of them had comorbid major depressive disorder (MDD), 3 had comorbid generalized anxiety disorder (GAD), and 1 had both MDD and GAD comorbidities). 170 trauma-exposed adults met no diagnostic criteria for mental illness or substance-use disorders. This study excluded 10 trauma-exposed adults diagnosed with other psychiatric disorders (5 of them had MDD, 4 had GAD, and 1 had both generalized anxiety and MDD).

After the subsequent MRI scanning, the following conditions were excluded: any history or current brain injury or any other major medical or neurological conditions (5 trauma-exposed adults without PTSD were ruled out due to these indications: 4 of them had cerebral ischemia or infarction and 1 had MDD and its associated antidepressants), and left-handedness (none).

### Measures

Every bereaved adult was evaluated using a suite of neuropsychological assessments, which included: the Hamilton Depression (HAMD) [23], Hamilton Anxiety (HAMA) [24] rating scales and the Mini-Mental State Examination (MMSE) [25]. HAMD and HAMA are the most commonly used scale to assess depression and anxiety state in clinic. There are 17 items in total. The more serious the disease, the higher the total score. If the total score is less than seven, it is normal. MMSE is the most widely used cognitive function screening tool at home and abroad. There are 30 items in total, and the total score range of the scale is 0–30 points. The higher the total score, the higher the cultural level.

### DNA genotyping

Of all the participants enrolled in this study, three trauma-exposed adults without PTSD refused to undergo the blood collection procedure. All other subjects provided peripheral blood samples, from which DNA material was extracted for further analysis. Genesky Biotechnologies, Inc. (Shanghai, China) [26] developed the Improved Multiple Ligase Detection Reaction (iMLDR) technique for genotyping *SLC6A2* rs2242446 (detailed primers were listed in Supplementary Table S1). iMLDR is an improved multiple SNP typing technology which uses PCR products as templates for high-specific double ligation reaction. Randomly selected sample from 5% of the samples were confirmed, and their results were 100% concordant. Deviation of genotype distributions from the Hardy Weinberg equilibrium (HWE) was assessed with the χ^2^ test for goodness of fit. HWE means that for a large and randomly mated population, the allele frequency and genotype frequency will remain unchanged without migration, mutation and selection. If the genotype distribution of a SNP locus in the study does not conform to HWE, the SNP typing data cannot be analyzed.

### Image acquisition

We used a 3.0T Philips MR scanner (Achieva 3.0 TTX; Amsterdam, the Netherlands) to acquire high-resolution structural MR images. The head motion was minimized during the acquisition of the images by applying foam pads. While scanning, participants were told to remain awake, close their eyes, and hold still. Three-dimensional T_1_-weighted structural brain images were acquired in the sagittal orientation using the turbo fast echo (3D-T1TFE) sequence: repetition time (TR)/echo time (TE) = 9.7/4.6; flip angle = 9°; matrix size = 256 × 256; field of view (FOV) = 256 × 256 mm^2^; slice thickness = 1 mm; number of slices = 160.

### Structural data preprocessing

Our preprocessing toolbox used SPM12 (http://www.fil.ion.ucl.ac.uk/spm) and the CAT12 (http://dbm.neuro.uni-jena.de/cat12/) voxel-based morphometry (VBM) for T1-weighted structural brain images. All parameters have been set to the default values recommended by the CAT12 manual, with the exception of the template option, which has been set to affine regularization with the International Consortium for Brain Mapping template for East Asian brains [27]. Then DARTEL algorithm is used to correct the bias field, classify the tissue and normalize the space of the structure images. The images were then segmented into cerebrospinal fluid (CSF), white matter (WM), and gray matter (GM). Tissue deformation was performed to adjust the segmented GM images of participants. Finally, a Gaussian filter (8 mm full width at half maximum, FWHM) was used to smooth the normalized and modulated GM images. After performing the quality control, one participant (without PTSD) was excluded for poor image quality.

### Statistical analysis

The SPSS 26(IBM Corp, Armonk, NY, USA) software package was used to perform data analysis of the PTSD and non-PTSD groups. Two sample t-test (two tailed, *P* < 0.05) and chi square test were used to explore differences in demographic and scoring data.

For GM data, we used SPM12 for statistical analysis. A 2 × 3 full factorial model, with diagnosis and genotype (TT, CT, and CC) as independent variables, was used to evaluate morphological changes in GM. Age, gender, BMI, years of education, time duration since losing the only child and TIV were used as covariates to control for confounding variables. The 2 × 3 factorial designs were compared using the following t-tests: (a) diagnostic effect, for PTSD (TT, CT and CC) versus non-PTSD (TT, CT and CC); (b) *SLC6A2* genotype effect, for TT genotype participants (PTSD and non-PTSD) versus CT genotype participants (PTSD and non-PTSD) versus CC genotype participants (PTSD and non-PTSD); (c) *SLC6A2* genotype × PTSD diagnosis interaction, diagnosis effects in TT genotype individuals versus CT genotype individuals versus CC genotype individuals. These analyses resulted in a statistical parameter map {SPM (F)} based on the voxel level height threshold of *P* < 0.001. Gaussian random field (GRF) correction was performed on the resultant parameter map. The significance threshold was set at *P* < 0.05 which corresponded to a voxel *P* < 0.005 and a cluster level with *P* < 0.05. A Pearson’s correlation analysis was used to investigate the association between GMV showing significant effects (after ANOVA analysis) and diagnostic outcomes from the CAPS, HAMD, and HAMA assessments.

## Results

### Sample demographics

After removing individuals through exclusion criteria and genomic data availability, a total of 218 Han Chinese adults who lost their sole child were included for the subsequent analyses in our study. High-quality imaging data, neuropsychological test scores and *SLC6A2* genotype information have been collected for this group. The distribution of the *SLC6A2* genotypes was found to be in HWE (χ2 = 3.229, *P* > 0.05). Demographic data and neuropsychological tests scores for our study participants are summarized in Tables 1 and 2.

**Table 1:** Demographics of study participants: Han Chinese adults who lost an only child

Protocols	Adults with PTSD (n = 57)	Adults without PTSD (n = 161)	*P* value
Age, y	57.58 ± 5.48	58.80 ± 5.51	0.15^a^
Gender (F/M)	40/17	71/90	0.001^b^
Education, y	6.42 ± 4.18	6.71 ± 3.56	0.62^a^
BMI	24.35 ± 2.44	24.28 ± 2.82	0.88
Duration since trauma, m	57.35 ± 48.44	108.16 ± 71.71	< 0.001^a^
CAPS_total	47.65 ± 12.84	16.14 ± 10.02	<0.001^a^
HAMD	15.93 ± 6.61	5.86 ± 4.24	<0.001^a^
HAMA	12.65 ± 6.52	4.57 ± 3.45	<0.001^a^

Values are expressed as mean ± standard deviation. PTSD, post-traumatic stress disorder; BMI, body mass index; CAPS, clinician-administered PTSD scale; HAMD, Hamilton Depression; HAMA, Hamilton Anxiety

^a^ The *P* values for the difference between the two trauma-exposed groups was obtained by performing a two-sample t-test

^b^ The *P* values for gender distribution between the two trauma-exposed groups was obtained by performing a chi-square test

**Table 2:** Demographic data of *SLC6A2* genotypic subgroups for epistatic effect analysis

Protocols	PTSD(n = 57)			non-PTSD(n = 161)		
	TT(n = 28)	CT(n = 19)	CC(n = 10)	TT(n = 75)	CT(n = 66)	CC(n = 20)
Genotype frequency	49.1%	33.3%	17.6%	46.6%	41.0%	12.4%
Gender (F/M)	17/11	14/5	9/1	32/43	31/35	8/12
Age, y	59.25 ± 4.41	56.58 ± 6.10	54.8 ± 5.06	59.59 ± 5.43	58.41 ± 5.48	57.1 ± 5.27
Education, y	6.54 ± 3.77	6.16 ± 4.51	6.6 ± 4.41	6.28 ± 3.44	6.91 ± 3.54	7.65 ± 3.73
Duration since trauma, m	58.39 ± 42.84	68.84 ± 57.45	32.6 ± 29.43	111.52 ± 71.67	110.59 ± 74.53	87.5 ± 55.36
CAPS_total	47 ± 13.29	46.32 ± 12.26	52 ± 10.91	15.89 ± 9.77	16.38 ± 10.96	16.25 ± 6.92
HAMD	14.89 ± 6.43	15.58 ± 6.21	19.5 ± 6.31	5.62 ± 3.86	6.23 ± 4.61	5.53 ± 4.08
HAMA	13.07 ± 8.06	11.84 ± 4.96	13 ± 2.79	4.73 ± 3.45	4.66 ± 3.6	3.68 ± 2.58

Data are presented as mean ± standard deviation. PTSD, adults with PTSD; non-PTSD, adults without PTSD; BMI, body mass index; CAPS, clinician-administered PTSD scale; HAMD, Hamilton Depression Scale; HAMA, Hamilton Anxiety Scale

### VBM analysis

**Fig. 1:**
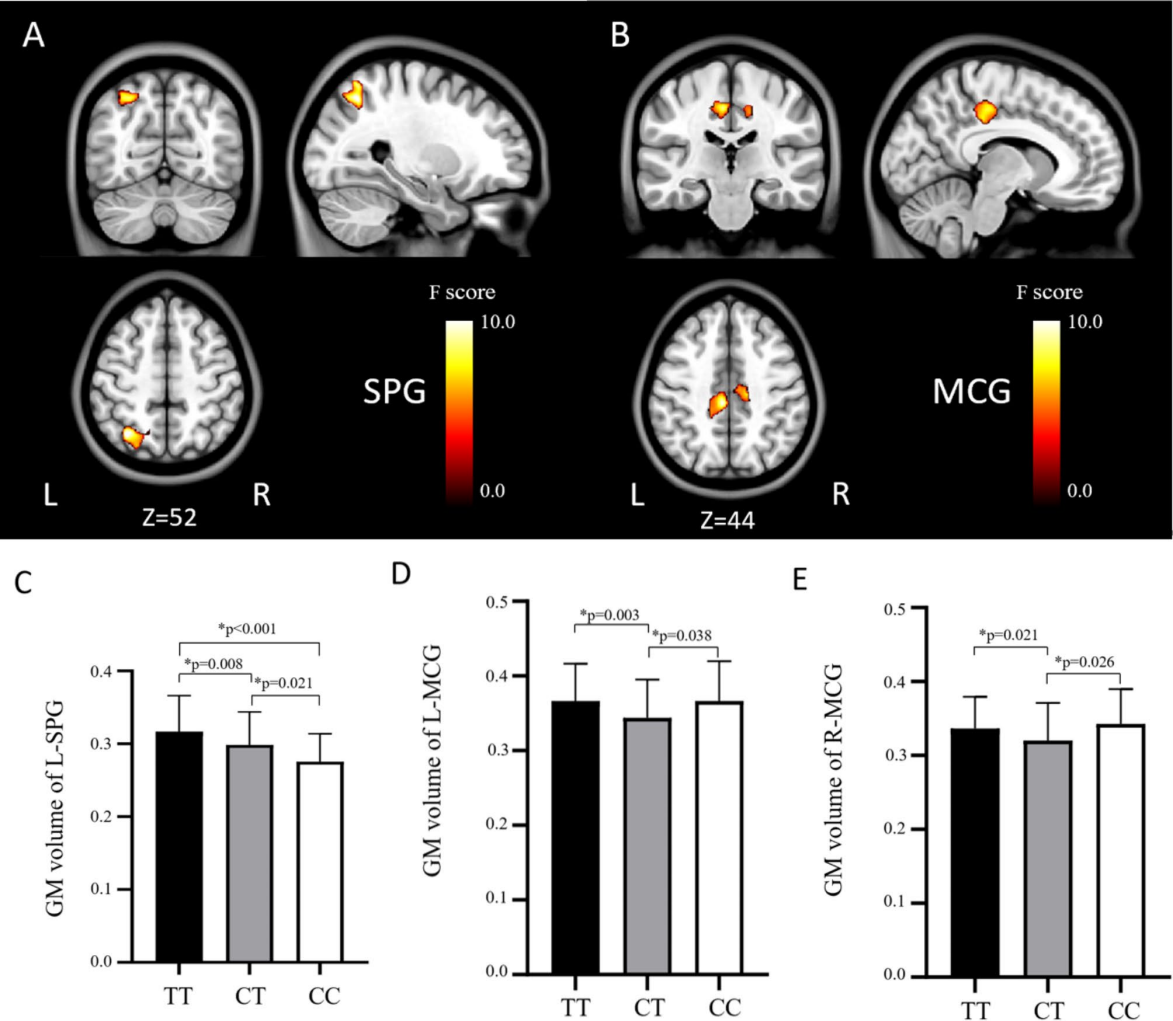
Brain regions with a significant *SLC6A2* genotype effects on brain GMV (*P* < 0.05, GRF corrected). Color bars show *F* scores. Bar plots depict the mean values and standard error of GMV in each genotypic subgroup. The asterisks * represent significant GMV difference between genotypic subgroups (*P* < 0.05). GM, gray matter; L, left; R, right; SPG, superior parietal gyrus; MCG, middle cingulate gyrus

No significant main effects for diagnosis were found in this study. Significant *SLC6A2* genotype effects on GMV (*P* < 0.05, GRF corrected) were found in the left superior parietal gyrus (L-SPG) and the bilateral middle cingulate gyrus (MCG) (Fig. 1). The *SLC6A2* CC genogroup had a significantly smaller GMV (*P* < 0.05) in the left superior parietal gyrus than the other two genogroups. Furthermore, the *SLC6A2* CC genogroup also exhibited significantly larger GMV in the bilateral middle cingulate gyrus than the CT genogroup. No significant GMV differences in the bilateral middle cingulate gyrus were found between the *SLC6A2* CT and TT genotype participants (*P* > 0.05, uncorrected).

**Fig. 2:**
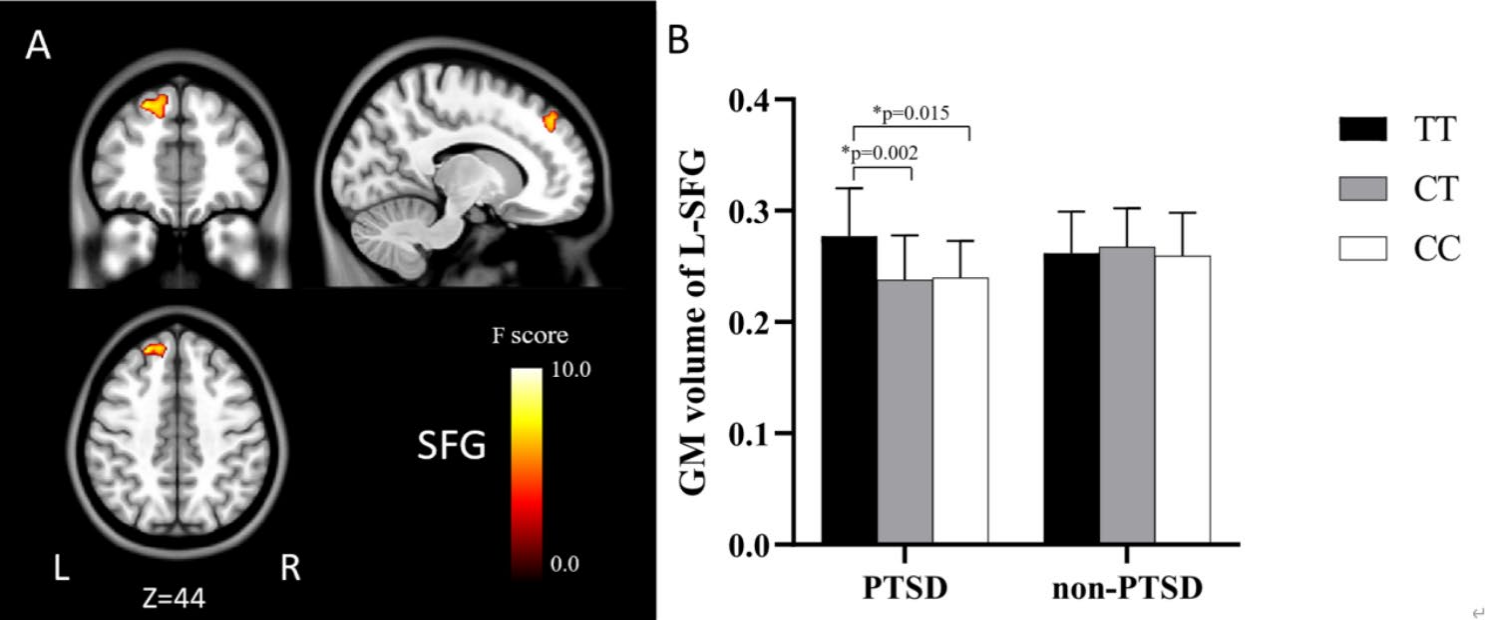
Brain regions with a significant *SLC6A2* genotype × diagnosis interaction on brain GMV (*P* < 0.05, GRF corrected). Color bars show *F* scores. Bar plots depict the mean values and standard error of GMV in each genotypic subgroup. The asterisks * represent significant GMV difference between genotypic subgroups (*P* < 0.05). GM, gray matter; L, left; R, right; SFG, superior frontal gyrus

Significant *SLC6A2* genotype-diagnosis interaction effects were found in the left superior frontal gyrus (L-SFG) (GRF corrected *P* < 0.05) (Fig. 2). Within the PTSD group, *SLC6A2* TT homozygous carriers had larger left superior frontal gyrus volumes than the CT and CC carriers (*P* < 0.05); however, in the non-PTSD group, no significant differences were found among the three genogroups (*P* > 0.05).

### Correlation analysis

There was no significant correlation between brain areas showing a significant genotype main effect, diagnosis × *SLC6A2* interaction, and CAPS, HAMA, or HAMD scores.

## Discussion

In the study, we researched the effects of PTSD diagnosis, *SLC6A2* polymorphism and PTSD diagnosis × *SLC6A2* genotype interaction on GMV in Han Chinese adults who lost their sole child. We found the main effect of *SLC6A2* polymorphism on superior parietal gyrus (SPG) and middle cingulate gyrus (MCG) volumes, and the interaction effect of PTSD diagnosis × *SLC6A2* genotype on superior frontal gyrus (SFG) volume.

In our study, we did not find the main effect of PTSD diagnosis; which is to say that there was no significant difference in GMV between the PTSD group and the non-PTSD group. The variability in structural findings across PTSD studies could arise from differences within the sample population with regard to trauma types [28]. This is in alignment with previous studies, which also found no significant brain volume changes between PTSD and non-PTSD adults who lost their sole child [29–31].

One main finding in this study was the effect of the *SLC6A2* genotype on regional GMV. We found a pattern showing that individuals who possess more C alleles in their *SLC6A2* genotype have decreased GMV at their L-SPG region. In other words, the three genogroups show the following trend for GMV at L-SPG (in descending order): TT genotype > CT genotype > CC genotype. SPG is an important region involved in the integration of somatosensory and visual space perception, and plays an additional role in attention, written language and working memory [32]. Previous studies have shown that the C allele in the *SLC6A2* gene is associated with higher NET transcriptional activity and lower NE levels in the synaptic cleft. This is due to the C allele’s ability to enhance transcriptional activity of the *SLC6A2* gene by inactivating the repressor element in the promoter [33]. Low levels of NE can impair neuronal differentiation because NE induces the expression of brain-derived neurotrophic factor, the most common growth factor of the central nervous system [34, 35]. For this reason, we speculate that the decrease of NE level due to active re-uptake of NE in *SLC6A2* CT and CC genotype participants is related to the decrease of SPG volume. Meanwhile for MCG, this linear relationship between brain volume and number of risk C alleles was not detected in this study. An inverted U-shaped regulatory effect that catecholamines, including dopamine and NE, has a cognitive function in attention deficit hyperactivity disorder patients has been proposed in previous studies [36]. Thus, we speculate that, unlike SPG, the cingulate gyrus may be subject to the nonlinear regulatory effect of NE. However, this conjecture needs to be verified in future researches.

Another interesting finding in this study was the interaction effect of diagnosis × *SLC6A2* on the brain volumes. Specifically, PTSD patients with the *SLC6A2* TT genotype had a greater GMV of L-SFG compared with PTSD patients with the CT genotype or CC genotype. The SFG, including the dorsolateral prefrontal cortex, is an important brain region. It is generally considered to be the core area of advanced cognitive function, including attention, working memory, cognitive control, motivational behavior and emotional regulation [37]. Studies have shown that GMV reduction in frontal lobe regions, including the SFG, is common in patients with PTSD [38–40]. Furthermore, this reduction in volume is potentially associated with the sustained vigilance and alertness associated with PTSD [39]. The NE system’s main function involves regulating alertness, while it also projects to various cortical regions including the frontal lobe [36]. Therefore, we reason that the differences in *SLC6A2* gene expression could have an influence on the volume of frontal gray matter in patients with PTSD, through affecting the amount of NE production and leading to PTSD-related alertness symptoms. Chronic stress has been shown to up-regulate the expression of *SLC6A2* gene in the locus coeruleus, hippocampus, frontal cortex and amygdala, and significantly increases NET protein levels [41]. This forms the basis of our speculation that SNP differences in the *SLC6A2* gene may be an important underlying factor behind the pathophysiological mechanisms for the occurrence and progression of PTSD. However, in patients with PTSD, we did not find a correlation between the severity of PTSD symptoms and *SLC6A2* genotypes, so further research is warranted to investigate this interpretation.

Interestingly, we observed differences only in the left SPG and SFG, not in the right. A possible explanation for these results is that the SLC6A2 gene may have a non-uniform regulation of gray matter volume across the cortex. Our observation may potentially be corroborated by findings from others: Meyer et al. found that state-dependent changes in frontal asymmetry could serve as a biological marker of PTSD symptoms [42]. Luo et al. also found that the hippocampal volume deficits showed patterns of laterality; the left side was affected more than the right in PTSD patients [43]. In summary, further research is needed to investigate this potential laterality in the regulation of PTSD gray matter volume by SLC6A2 genes.

Our investigation has several limitations. First, since our study was a small survey research and the participants were difficult to obtain, the sex ratio between groups was unbalanced. We may need a larger sample size with a balanced sex ratio of PTSD patients who lost their only child to verify our results in the future. What’s more, some other structural measurements like surface thickness or structural covariance, should also be explored in the future researches. Second, the findings from this study should be considered provisional and preliminary. The implications should be interpreted with caution, until they can be replicated in larger samples or validated by GWAS findings on PTSD. Third, since our research focuses on the specific subpopulation of Chinese adults who have lost their sole child, additional discretion should be employed when choosing to generalize these results to other population groups. Forth, in addition to *SLC6A2*, there may be other genetic or environmental factors that affect the brain norepinephrine system and the SPG volume of PTSD patients, that are not currently within the scope of our hypothesis.

Future large-scale work would benefit from employing designs with a more balanced gender ratio, recruiting PTSD patients who experienced other types of stressors, and incorporating possible environmental factors. These may contribute to a more comprehensive exploration or verification of the impact of SLC6A2 polymorphism on GMV in PTSD patients.

## Conclusions

In this study, we found that rs2242446 SNPs of the *SLC6A2* gene regulates the association between PTSD diagnosis and gray matter volume in the superior frontal gyrus. This may help improve the current understanding of the key role that the *SLC6A2* gene plays in the pathogenesis of PTSD after experiencing the loss of a sole child.

### Electronic supplementary material

Below is the link to the electronic supplementary material.

Supplementary Material 1

## Data Availability

The datasets used and/or analysed during the current study available from the corresponding author on reasonable request.

## References

[1] Zeng Y, Zhang X, Liu L (2017). From selective two-child policy to universal two-child policy: will the payment crisis of China’s pension system be solved?. Finance & Trade Economics.

[2] Zheng Y, Lawson TR, Anderson Head B (2017). Our only child has Died-A study of Bereaved Older Chinese Parents. Omega (Westport) Mar.

[3] Wei Y, Jiang Q, Gietel-Basten S (2016). The well-being of bereaved parents in an only-child society. Death Stud.

[4] Song Y (2014). Losing an only child: the one-child policy and elderly care in China. Reprod Health Matters.

[5] Yin Q, Shang Z, Zhou N (2018). An investigation of physical and mental health consequences among Chinese parents who lost their only child. BMC Psychiatry Feb.

[6] Michopoulos V, Vester A, Neigh G (2016). Posttraumatic stress disorder: a metabolic disorder in disguise?. Exp Neurol Oct.

[7] Wang Q, Xu W, Ren L, Wang W, Wang Y (2019). The relationship between hope and post-traumatic stress disorder in Chinese shidu parents: the mediating role of perceived stress. J Affect Disord May.

[8] Duncan LE, Ratanatharathorn A, Aiello AE (2018). Largest GWAS of PTSD (N = 20 070) yields genetic overlap with schizophrenia and sex differences in heritability. Mol Psychiatry Mar.

[9] Nisar S, Bhat AA, Hashem S et al. Genetic and neuroimaging approaches to understanding post-traumatic stress disorder. Int J Mol Sci Jun 24 2020;21(12). 10.3390/ijms21124503 PMC7352752 32599917

[10] Nwokafor C, Serova LI, Tanelian A, Nahvi RJ, Sabban EL (2021). Variable response of Norepinephrine Transporter to traumatic stress and relationship to Hyperarousal. Front Behav Neurosci.

[11] Hein L (2006). Adrenoceptors and signal transduction in neurons. Cell Tissue Res.

[12] Gannon M, Wang Q (2019). Complex noradrenergic dysfunction in Alzheimer’s Disease: low norepinephrine input is not always to blame. Brain Res Jan.

[13] Mandela POG (2006). The norepinephrine transporter and its regulation. J Neurochem.

[14] Mayer AFSC, Heusser K (2006). Influences of norepinephrine transporter function on the distribution of sympathetic activity in humans. Hypertension.

[15] Bönisch HBM. The Norepinephrine Transporter in Physiology and Disease. Handb Exp Pharmacol 2006:485–524. 10.1007/3-540-29784-7_20 16722247

[16] Ordway GASC, Cason GW, Klimek V (1997). Pharmacology and distribution of norepinephrine transporters in the human locus coeruleus and raphe nuclei. J Neurosci.

[17] Oh SY, Kim YK. Association of norepinephrine transporter gene polymorphisms in attention-deficit/hyperactivity disorder in Korean population. Prog Neuropsychopharmacol Biol Psychiatry Oct 27 2016;S0278-5846(16)30326-8. 10.1016/j.pnpbp.2016.10.006 27984158

[18] Pietrzak RH, Sumner JA, Aiello AE (2015). Association of the rs2242446 polymorphism in the norepinephrine transporter gene SLC6A2 and anxious arousal symptoms of posttraumatic stress disorder. J Clin Psychiatry Apr.

[19] Young DA, Chao LL, Zhang H (2021). Ventromedial and insular cortical volume moderates the relationship between BDNF Val66Met and threat sensitivity. J Psychiatr Res Oct.

[20] Fujii TOM, Hori H (2014). Association between the common functional FKBP5 variant (rs1360780) and brain structure in a non-clinical population. J Psychiatr Res.

[21] Hayes JP, Logue MW, Reagan A (2017). COMT Val158Met polymorphism moderates the association between PTSD symptom severity and hippocampal volume. J Psychiatry Neurosci Mar.

[22] Spitzer R, First M, Williams J, Gibbon M. Structured clinical interview for DSM-IV-TR Axis I disorders, Research Version, Patient Edition. SCID-I/P); 2002.

[23] A rating scale for depression MH (1960). J Neurol Neurosurg Psychiatry.

[24] The assessment of anxiety states by rating MH (1959). Br J Med Psychol.

[25] The mini-mental state examination MF (1983). Arch Gen Psychiatry.

[26] Liu Y, Hu C, Liu C (2019). A rapid improved multiplex ligation detection reaction method for the identification of gene mutations in hereditary hearing loss. PLoS ONE.

[27] Liu S, Li A, Liu Y (2020). Polygenic effects of schizophrenia on hippocampal grey matter volume and hippocampus-medial prefrontal cortex functional connectivity. Br J Psychiatry May.

[28] Hinojosa CA (2022). Does hippocampal volume in patients with posttraumatic stress disorder vary by trauma type?. Harv Rev Psychiatry Mar-Apr.

[29] Luo Y, Shan H, Liu Y (2016). Decreased left hippocampal volumes in parents with or without posttraumatic stress disorder who lost their only child in China. J Affect Disord Jun.

[30] Luo Y, Liu Y, Qin Y (2017). The atrophy and laterality of the hippocampal subfields in parents with or without posttraumatic stress disorder who lost their only child in China. Neurol Sci Jul.

[31] Qi R, Luo Y, Zhang L (2020). Decreased functional connectivity of hippocampal subregions and methylation of the NR3C1 gene in Han Chinese adults who lost their only child. Psychol Med Jan.

[32] Lin YH, Dadario NB, Hormovas J (2021). Anatomy and White Matter connections of the Superior Parietal Lobule. Oper Neurosurg (Hagerstown) Aug.

[33] Yang X, Ru W, Wang B (2016). Investigating the genetic basis of attention to facial expressions: the role of the norepinephrine transporter gene. Psychiatr Genet Dec.

[34] Tully KBV. Emotional enhancement of memory how norepinephrine enables synaptic plasticity. Mol Brain 2010;3. 10.1186/1756-6606-3-15 PMC2877027 20465834

[35] Chen MJNT, Pike CJ, Russo-Neustadt AA (2007). Norepinephrine induces BDNF and activates the PI-3K and MAPK cascades in embryonic hippocampal neurons. Cell Signal.

[36] Nemoda Z, Angyal N, Tarnok Z (2019). Differential Genetic Effect of the Norepinephrine transporter promoter polymorphisms on attention problems in clinical and non-clinical samples. Front NeuroSci.

[37] Kraljevic N, Schaare HL, Eickhoff SB (2021). Behavioral, anatomical and Heritable Convergence of Affect and Cognition in Superior Frontal Cortex. Neuroimage Nov.

[38] Geuze E, Westenberg HG, Heinecke A, de Kloet CS, Goebel R, Vermetten E (2008). Thinner prefrontal cortex in veterans with posttraumatic stress disorder. Neuroimage Jul.

[39] O’Doherty DCM, Tickell A, Ryder W (2017). Frontal and subcortical grey matter reductions in PTSD. Psychiatry Res Neuroimaging Aug.

[40] Roy O, Levasseur-Moreau J, Renauld E (2022). Whole-brain morphometry in Canadian soldiers with posttraumatic stress disorder. Ann N Y Acad Sci Mar.

[41] Chen PFY, Li Y, Sun Z, Bissette G, Zhu MY (2012). Chronic social defeat up-regulates expression of norepinephrine transporter in rat brains. Neurochem Int.

[42] Meyer T, Quaedflieg CWEM, Weijland K, Schruers K, Merckelbach H, Smeets T. Frontal EEG asymmetry during symptom provocation predicts subjective responses to intrusions in survivors with and without PTSD. Psychophysiology. 2018;55(1). 10.1111/psyp.12779 28295402

[43] Luo Y, Liu Y, Qin Y, Zhang X, Ma T, Wu W, Yang Y, Jiang D, Shan H, Cao Z (2017). The atrophy and laterality of the hippocampal subfields in parents with or without posttraumatic stress disorder who lost their only child in China. Neurol Sci.

