# Supplementary material for: Relationship between SLC6A2 gene polymorphisms and brain volume in Han Chinese adults who lost their sole child
Source: BMC Psychiatry. 2024 Jan 2;24:11. doi: 10.1186/s12888-023-05467-4 (PMC10763183; doi:10.1186/s12888-023-05467-4)
Supplement: Supplementary file 1 — Supplementary Material 1 [file 12888_2023_5467_MOESM1_ESM.docx]

**Supplementary Table**

**Table S1.** Primer sequences for the *SLC6A2* SNPs

| SNP | Chromosome position | PCR Primer |  |
| --- | --- | --- | --- |
| Rs2242446 | 55690425^a^ | Forward | CTGCAGGGTCTTCAGCCG |
|  |  | Reverse | CTGAGCGGACGCAGGGTT |

^a^ Reference Genome: hg19

*SLC6A2* = solute carrier family 6 member 2; SNP = single nucleotide polymorphism; PCR = Polymerase chain reaction.
